# Coverage for Opioid Use Disorder Medications in Medicaid Managed Care

**DOI:** 10.1001/jamahealthforum.2025.3239

**Published:** 2025-09-05

**Authors:** Christina M. Andrews, Sage R. Feltus, Constance M. Horgan, Cindy Parks Thomas, Dominic Hodgkin, Maureen T. Stewart

**Affiliations:** 1 Department of Health Services Policy and Management, Arnold School of Public Health, University of South Carolina, Columbia; 2 Department of Health Law, Policy and Management, School of Public Health, Boston University, Boston, Massachusetts; 3 Heller School for Social Policy and Management, Brandeis University, Waltham, Massachusetts

## Abstract

This cross-sectional study describes the distribution of state Medicaid managed care plans providing and managing opioid use disorder medication benefits to beneficiaries in 2023.

## Introduction

Medicaid managed care plans (MCPs) play a critical role in facilitating access to treatment for opioid use disorder (OUD).^1^ The 2018 Substance Use Disorder Prevention that Promotes Opioid Recovery and Treatment for Patients and Communities (SUPPORT) Act required Medicaid MCPs to cover all medications for OUD (MOUD)—buprenorphine, extended-release naltrexone, and methadone—for 5 years beginning October 1, 2020.^2^ To our knowledge, no study has assessed the extent to which MCPs report MOUD coverage—defined as inclusion in member materials—consistent with the SUPPORT Act.

## Methods

All comprehensive MCPs offering coverage to adult Medicaid enrollees aged 18 to 64 years in 2023 and operating in a state in which MCPs are responsible for managing OUD medication benefits were identified from state Medicaid websites. MCP-preferred drug lists (which detail coverage of buprenorphine and naltrexone) and member handbooks (which include information on methadone coverage) were obtained from plan websites in December 2023 (eMethods in Supplement 1). In accordance with the Common Rule, this cross-sectional study was exempt from ethics review and informed consent because it was not considered human participant research. We followed the STROBE reporting guideline.

Descriptive statistics were calculated to examine MCP-reported coverage of each medication and all MOUD combined. We also calculated, by state, the total number and percentage of Medicaid enrollees served by MCPs covering each medication and all MOUD combined. We mapped the percentage of MCPs that reported covering each medication and all MOUD by state. States included or excluded in analysis are listed in eTable in Supplement 1. Data were analyzed from November 2024 to January 2025 using Stata, version 18.0 (StataCorp).

## Results

There were 180 comprehensive MCPs in 31 states and Washington, DC, covering 37.1 million enrollees in 2023. Among these MCPs, 175 (97.2%) with 36.5 million enrollees (98.4% of total enrollees) reported covering buprenorphine, 143 (79.4%) with 31.8 million enrollees (84.8%) covered extended-release naltrexone, and 85 (47.2%) with 19.6 million enrollees (52.8%) covered methadone. Sixty-seven MCPs (37.2%) with 17.3 million enrollees (46.6%) reported covering all MOUD (Table). In 10 states, no MCPs reported covering all MOUD (Figure).

**Table.** Distribution of Medicaid MCPs Covering Opioid Use Disorder Medications in 2023

	Medicaid MCPs, No. (%)				
	Total (N = 185)	Reported covering selected medications			
		Buprenorphine	Naltrexone^a^	Methadone	All MOUD
Total plans	180 (100)	175 (97.2)	143 (79.4)	85 (47.2)	67 (37.2)
Total enrollees, in millions	37.1 (100)	36.5 (98.4)	31.8 (84.8)	19.6 (52.8)	17.3 (46.6)

Abbreviations: MCP, managed care plan; MOUD, medications for opioid use disorder.

^a^ Naltrexone represents extended-release naltrexone.

**Figure.**
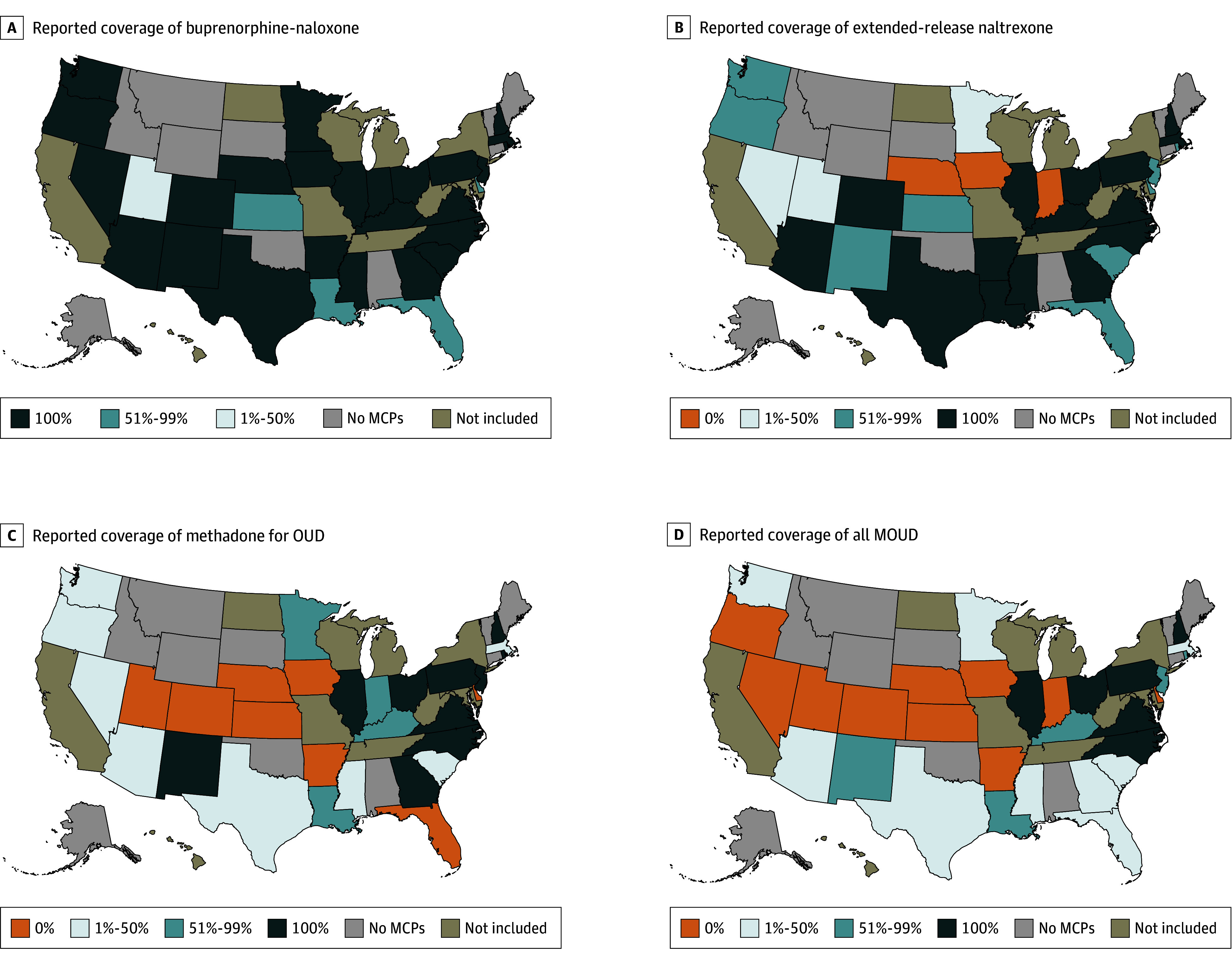
Percentage of Comprehensive Medicaid Managed Care Plans (MCPs) Reporting Coverage of Opioid Use Disorder (OUD) Medications by State in 2023 MOUD indicates medications for OUD.

## Discussion

The findings of this study reveal widespread gaps in Medicaid MCP publicly reported coverage of OUD medications in 2023, despite the federal requirement in the SUPPORT Act that all state Medicaid programs—and, by extension, their contracted MCPs—provide coverage for all 3 MOUD by October 1, 2020. Inconsistency between Medicaid MCP–reported coverage and federal regulations may not reflect the considered decision by Medicaid plans to ignore the law. Delays in implementation of legislation are common,^3^ and it is possible that our findings reflect an inability to institute policy change in a timely manner. It is also possible that MCPs do cover all MOUD but simply do not report coverage in their public-facing materials. However, if MCPs do not specify which MOUD are covered, enrollees will be less likely to access the medications.

A study limitation is MCPs may cover MOUD but not report it in their materials. Additionally, the findings do not generalize to plans in states where the state retains the responsibility for MOUD.

MOUD are lifesaving.^4^ Efforts to increase use of MOUD are less effective when insurance barriers exist. Further research is needed to understand the extent of noncompliance with federal requirements and the underlying factors in this phenomenon.

Medicaid MCPs should review their benefits materials to ensure these documents communicate all covered services and comply with federal law. State Medicaid agencies should be explicit regarding federal requirements in the SUPPORT Act and the Medicaid Drug Rebate Program and specify these policies in plan contracts. The Centers for Medicare & Medicaid Services should clarify the consequences of noncompliance for MCPs and the states with which they contract. The 2025 reauthorization of the SUPPORT Act provides a crucial opportunity to address the noncompliance problem.

## Notes

Supplement 1. **eMethods.** Identifying Methadone Coverage **eTable.** US States Included and Excluded in Analysis

Supplement 2.
Data Sharing Statement
