# Supplementary material for: Coverage for Opioid Use Disorder Medications in Medicaid Managed Care
Source: JAMA Health Forum. 2025 Sep 5;6(9):e253239. doi: 10.1001/jamahealthforum.2025.3239 (PMC12413645; doi:10.1001/jamahealthforum.2025.3239)
Supplement: Supplement 1. — eMethods. Identifying Methadone Coverage eTable. US States Included and Excluded in Analysis [file jamahealthforum-e253239-s001.pdf]

Supplemental Online Content

Andrews CM, Feltus SR, Horgan CM, Thomas CP, Hodgkin D, Stewart MT. Coverage for opioid use disorder medications in Medicaid managed care. *JAMA Health Forum*. 2025;6(9):e253239. doi:10.1001/jamahealthforum.2025.3239

eMethods. Identifying Methadone Coverage

eTable. US States Included and Excluded in Analysis

This supplemental material has been provided by the authors to give readers additional information about their work.

© 2025 Andrews CM et al. *JAMA Health Forum*

eMethods. Identifying Methadone Coverage

Methadone for the treatment of opioid use disorder is typically covered by managed care plans as a service under the medical benefit and was coded as covered by the plans if the member handbook listed coverage for any of the following: opioid treatment programs, methadone maintenance, or opioid substitution treatment. As of 2023 in the United States, opioid treatment programs are the only healthcare facilities that provide methadone for opioid use disorder, with few exceptions. The terms “methadone maintenance” and “opioid substitution treatment” are commonly used to refer to methadone treatment for opioid use disorder.

© 2025 Andrews CM et al. *JAMA Health Forum*

eTable. US States Included and Excluded in Analysis

Excluded			Included
**State does not offer any comprehensive Medicaid managed care (n=10 states)**	**State retains pharmacy benefit for substance use disorder treatment (n=9 states)**	**State was exempt from the SUPPORT Act requirements (n=1 state)**	**Medicaid managed care plans in state responsible for pharmacy benefit for substance use disorder treatment (n= 31 states and District of Columbia)**
Alabama, Alaska, Connecticut, Idaho, Maine, Montana, Oklahoma, South Dakota, Vermont, Wyoming	New York, Tennessee, West Virginia, California, Maryland, Michigan, Missouri, North Dakota, Wisconsin	Hawaii	Arkansas, Arizona, Colorado, Delaware, Florida, Georgia, Iowa, Illinois, Indiana, Kansas, Kentucky, Louisiana, Massachusetts, Minnesota, Mississippi, North Carolina, Nebraska, New Hampshire, New Jersey, New Mexico, Nevada, New York, Ohio, Oregon, Pennsylvania, Rhode Island, South Carolina, Texas, Utah, Virginia, Washington, District of Columbia

© 2025 Andrews CM et al. *JAMA Health Forum*
