# Supplementary material for: Coverage for Opioid Use Disorder Medications in Medicaid Managed Care
Source: JAMA Health Forum. 2025 Sep 5;6(9):e253239. doi: 10.1001/jamahealthforum.2025.3239 (PMC12413645; doi:10.1001/jamahealthforum.2025.3239)
Supplement: Supplement 2. — Data Sharing Statement [file jamahealthforum-e253239-s002.pdf]

Data Sharing Statement

Andrews. Coverage for Opioid Use Disorder Medications in Medicaid Managed Care. *JAMA Health Forum*. Published September 05, 2025. doi:10.1001/jamahealthforum.2025.3239

Data
Data available: Yes
Data types: Data (not involving human participants)
How to access data: Data will be made available upon request. Please contact Dr. Maureen Stewart at stewartm@bu.edu.
When available: With publication

Supporting Documents
Document types: None

Additional Information
Who can access the data: Researchers whose proposed use of the data has been approved.
Types of analyses: The data will be made available for analyses reviewed by the study principal investigator (Stewart).
Mechanisms of data availability: After approval of a proposal and with a signed data access agreement.
